# Predicting the synergy of multiple stress effects

**DOI:** 10.1038/srep32965

**Published:** 2016-09-09

**Authors:** Matthias Liess, Kaarina Foit, Saskia Knillmann, Ralf B. Schäfer, Hans-Dieter Liess

**Affiliations:** 1 UFZ - Helmholtz Centre for Environmental Research, Dept. System-Ecotoxicology, Permoserstrasse 15, D-04318 Leipzig, Germany; 2 RWTH Aachen University, Institute for Environmental Research (Biology V), Worringerweg 1, 52074 Aachen, Germany; 3 University Koblenz-Landau, Institute for Environmental Sciences, Fortstrasse 7, 76829 Landau in der Pfalz, Germany; 4 University of the Bundeswehr München, Faculty EIT, W-Heisenberg-Weg 39, 85577 Neubiberg, Germany

## Abstract

Toxicants and other, non-chemical environmental stressors contribute to the global biodiversity crisis. Examples include the loss of bees and the reduction of aquatic biodiversity. Although non-compliance with regulations might be contributing, the widespread existence of these impacts suggests that for example the current approach of pesticide risk assessment fails to protect biodiversity when multiple stressors concurrently affect organisms. To quantify such multiple stress effects, we analysed all applicable aquatic studies and found that the presence of environmental stressors increases individual sensitivity to toxicants (pesticides, trace metals) by a factor of up to 100. To predict this dependence, we developed the “Stress Addition Model” (SAM). With the SAM, we assume that each individual has a general stress capacity towards all types of specific stress that should not be exhausted. Experimental stress levels are transferred into general stress levels of the SAM using the stress-related mortality as a common link. These general stress levels of independent stressors are additive, with the sum determining the total stress exerted on a population. With this approach, we provide a tool that quantitatively predicts the highly synergistic direct effects of independent stressor combinations.

Amongst a multitude of environmental stressors also toxicants are omnipresent in the environment and contribute to the global biodiversity crisis^1^. In global surface waters insecticide contamination is generally present and frequently exceeds the regulatory threshold levels^2^. This toxic pressure results in large-scale community alterations first identified in central Europe^3^, confirmed in several global regions^4^ and induce a reduction of aquatic biodiversity^5^. The community alterations occurred at pesticide concentrations 10 to 100 times lower than those predicted to be safe by governmental risk assessment frameworks. In addition, a similar discrepancy between observations in ecotoxicity test systems and wildlife has recently been identified for other anthropogenic stressors, including metals^6^ and ionizing radiation^7^.

Paracelsus was one of the first scientists to realize that the biological effects of a toxicant are amplified when an individual is weakened by additional unfavourable external factors, and 500 years ago, he asserted that such factors must be considered to understand and predict a toxicant’s effect (Paracelsus, Volumen Medicinae Paramirum). Since then, increasing evidence has suggested that the impacts of multiple stressors of different types may synergistically exceed the effects of individual stressors, as has been demonstrated for a range of organisms^8^. Examples include (i) wild bee populations and honey bee colonies, which have declined in response to the combined effects of pesticides and environmental stressors, such as parasites^9^, reduced floral abundance or weather^8^,^10^; (ii) marine crustaceans, which have exhibited an increased sensitivity to the combination of copper and ultraviolet radiation^11^; (iii) amphibians, which have been negatively affected by the interaction between agrochemicals and parasites^12^; and (iv) human populations, for which heat stress exacerbates the toxicity of many air pollutants, insecticides, and other toxic chemicals^13^.

In summary, requirements posed by additional environmental stressors, which are defined as any physical or biological entity that can induce an adverse response, reveal low-concentration effects of toxicants^14^,^15^. However, most stressor combinations relevant within the environment lack empirical evidence to derive threshold for combined risks. Therefore, the challenge of quantitatively predicting the combined impacts of stressors remains a fundamental issue in the medical and ecological sciences.

## Results

### A meta-analysis

**Figure 1:**
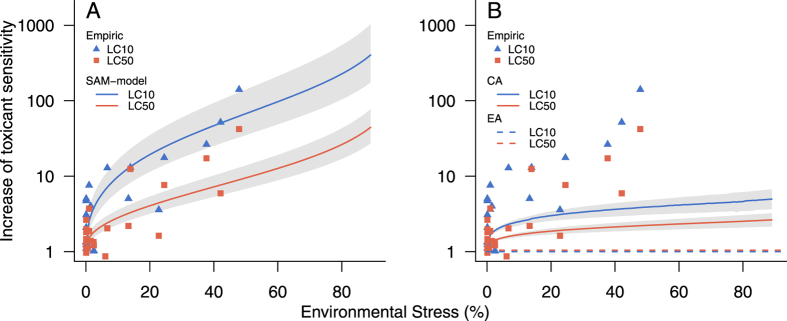
Increase of toxicant sensitivity in relation to additional environmental stress – (**A**) Stress Addition Model, SAM; (**B**) Traditional approaches from mixture toxicity. The x-axis shows the magnitude of environmental stress, expressed as mortality without toxicant exposure. The y-axis shows the increase in toxicant sensitivity. Observations: Points indicate aquatic vertebrate and invertebrate studies with 23 pairs of concentration-response relationships, including 6 different environmental stressors, 5 toxicants and 10 species. (**A**) Modelling by the SAM: Lines indicate the modelled increase in toxicant sensitivity for the LC10 (LC10/LC10*) and the LC50 (LC50/LC50*) in relation to the environmental stress. (**B**) Modelling by traditional approaches from mixture toxicity: Lines indicate the modelled increase in toxicant sensitivity using the extended approach of concentration addition (CA, blue and red solid lines), and effect addition (EA, blue and red dashed lines). Details on the experimental studies see Table S1 and Figure S1. The grey shaded area represents the results of the model based on the standard error of the average normalized concentration-response curve (see Methods).

We aimed to empirically identify the impact of additional environmental stress on the toxicant sensitivity of individuals. For this, we reviewed all published studies of the combined impact of environmental stressors and toxicants that met our inclusion criteria (see Methods, Table S1). This meta-analysis revealed that additional environmental stress strongly increases the toxicant sensitivity of individuals (Fig. 1). The increase of toxicant sensitivity is thereby quantified as the shift of the lethal concentration from the concentration-response-relationship of the toxicant alone (LCx) compared to the concentration-response relationship of the toxicant under environmental stress (LCx*). For high-effect levels of a toxicant (50% mortality; LC50/LC50*), the presence of environmental stressors increases individual sensitivity to toxicants by a factor of up to 10, whereas for low-effect levels of a toxicant (10% mortality; LC10/LC10*), the presence of environmental stressors increases individual sensitivity to toxicants by a factor of up to 100. It is also remarkable that even levels of environmental stress without a measurable mortality effect may considerably increase the sensitivity to toxicants (Fig. 1). The relationship between environmental stress and sensitivity to toxicants could be approximated by a linear regression (LC50/LC50* r^2^ = 0.65, p < 0.001; LC10/LC10* r^2^ = 0.63, p < 0.001). This close relationship is particularly surprising because the analysis included 6 different environmental stressors, 5 toxicants with 3 different modes of action and 10 different vertebrate and invertebrate species (Table S1). For the first time, this approach systematically quantified the increase of toxicant sensitivity in relation to environmental stress for several investigations across various stressors and species.

### The Stress Addition Model ‘SAM’

**Figure 2:**
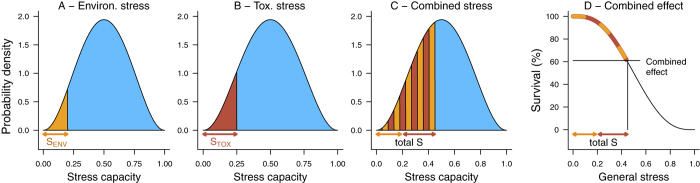
The Stress Addition Model (SAM). (**A,B**) Individual stress capacity within a population, beta-distributed. Under stress conditions, individuals with a stress capacity below the given stress level will die (**A**), orange area - environmental stress, S_ENV_; (**B**), red area - toxicant stress, S_TOX_) and individuals with a stress capacity above the present stress level will survive (**A** and **B**, blue area). (**C**) The total stress that is exerted on a population is calculated as the sum of single stress levels of independent stressors (orange and red areas). Only individuals with a stress capacity above the total stress will survive. (**D**) The joint effect expressed as a stress-related survival curve (see Methods for a mathematical description).

**Figure 3:**
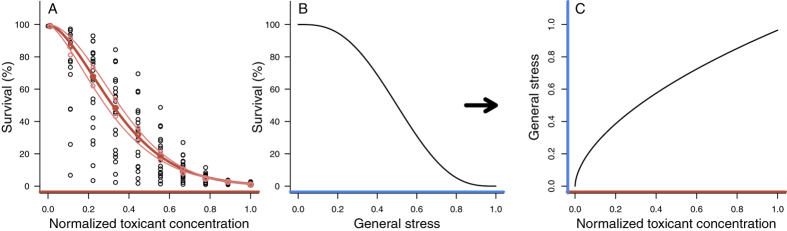
Transfer of toxicant concentration into general stress levels. (**A**) The normalized concentration-response curves from all investigated studies are depicted as points, the fitted concentration-response model for the average concentration-response curve as red line and the corresponding standard error as light red lines. (**B**) Shows the relationship of general stress levels and the population survival according to equation 4 of the SAM. (**C**) Identifies the resulting mortality-related link between concentration levels from the average normalized concentration-response curve and the general stress level. The relationship indicates that low toxicant concentrations disproportionally increase the general stress compared to high toxicant concentrations.

Based on the empirical observations, we developed the “Stress Addition Model” (SAM). This model relies on three principal assumptions that provide a mechanistic understanding of the combined impact of independent stressors, in this case a toxicant in combination with one environmental stressor. The mathematical details are described in the Methods section.

(1)The first assumption of the SAM is that each individual has a certain tolerance towards all types of stress, its general stress capacity. We assume that this individual stress capacity is beta distributed within a population following a bell-shaped curve within the interval of 0 to 1 (Fig. 2A). Under non-stress conditions, all individuals survive. Under stress conditions, individuals with a stress capacity below the given stress level will die (e.g., Fig. 2A/B, orange/red area) and individuals with a stress capacity above the present stress level will survive (e.g., Fig. 2A/B, blue area).

(2)The second assumption of the SAM is that every specific unit of a given stressor (e.g., μg/L for toxicants, °C for temperature) can be transferred into a general stress level ranging from 0 to 1 as a “common currency” for all stressors. A general stress level of 0 relates in no mortality, a general stress level of 1 results in 100% mortality. The transfer uses the stress-related mortality as a common link. As shown in Fig. 2A, the mortality induced by the environmental stressor S_ENV_ is related to the general stress level shown on the x-axis (orange area, general stress of 0.2). In Fig. 2B, the same is shown for the toxicant stressor S_TOX_ (red area, general stress of 0.25). Accordingly, the general stress level for each stressor can be identified with the observed mortality that is exerted by this stressor alone. For each stressor a transfer function can be obtained that links the observed mortality and the general stress level as illustrated in Fig. 3 and described in the Methods section.

(3) The third assumption of the SAM is that general stress levels of independent stressors are additive, with the sum determining the total stress exerted on a population (Fig. 2C, S_ENV_ + S_TOX_ = 0.2 + 0.25 = total stress S of 0.45). Hence, only individuals with a stress capacity higher than the total stress will survive (Fig. 2C, blue area). Figure 2D depicts the joint effect of two stressors as a stress-related survival curve.

### Application of the SAM

In a first analysis, we applied the SAM on each of the 23 experimental study-pairs of the meta-analysis separately (Figure S1, Table S1). For this we used the experimental information of (i) the concentration-response-relationship of the toxicant alone and (ii) the effect strength of the environmental stress that is additionally applied. With this information we predicted the concentration-response relationship of the toxicant under environmental stress using the SAM for the respective study (Figure S1, dashed lines). The final endpoint of the SAM is the predicted increase of toxicant sensitivity from the lethal concentration LCx without environmental stress to the lethal concentration LCx* with environmental stress. To quantify the goodness of model fit, we compared the observed and modelled shifts of LC10 (LC10/LC10*) and LC50 (LC50/LC50*) given as the proportion of variance explained by the model R^2^ (A:R^2^ = 0.49; B:R^2^ = 0.38).

In a final application of the SAM, we predicted the mean increase of toxicant sensitivity in relation to environmental stress over the whole data set of the meta-analysis (Fig. 1A, depicted as lines). For this we used (i) the normalized and averaged concentration-response-relationship over all studies of the meta-analysis (Fig. 3A) and (ii) assumed an increase of environmental stress levels from 1 to 90% mortality. The increase of toxicant sensitivity due to environmental stress is quantified as the shift of LC50 and LC10 and shows a good fit between the observed and predicted values (Fig. 1A). Additionally, we calculated the joint effects of two stressors applying traditional approaches from mixture toxicity, namely the concepts of concentration addition (CA) and effect addition (EA). The corresponding mathematical description is given in the Methods section. The results indicate that these predictions do not match the observations made in the meta-analysis of experimental studies (Fig. 1B). We reveal that both traditional approaches greatly under-predict the observed joint mortality in more than 1/2 of the experiments investigated.

## Discussion

The key challenge to predict multiple stress effects is to identify a “common currency” to quantify and join different independent stressors^16^. We solved this problem by assuming a universal capacity towards all types of stress, the general stress capacity within the SAM framework. This idea of a general stress capacity enabled us to transform all specific stressors into general stress levels. Applying this approach to the experimental studies published in the literature we were able to predict the combined effect of toxicants and additional environmental stressors. This ability however, is of fundamental relevance for prediction, assessment and management of anthropogenic stressors^17^,^18^. Based on a meta-analysis of existing literature, we revealed a key feature of combined stress effects: The presence of an additional environmental stressor caused disproportionally high mortality rates already at low concentrations of a toxicant when compared to the effect of the toxicant alone. It follows that the combined effects of independent stressors are highly synergistic compared to the results modelled applying the effect addition model (EA) and also the concentration addition model (CA). This observation that many stressor combinations may act synergistically has often been revealed^8^,^18^,^19^.

We argue that the SAM framework extends the traditional approaches of mixture toxicity. First, the approach of effect addition EA^20^ assumes toxicants with an independent effect mechanism. According to this concept, no stressor interaction is assumed. Hence, EA predicts no increased toxicant sensitivity when additional environmental stressors are present. This prediction does not match the observations made in the meta-analysis of experimental studies (Fig. 1B, dashed lines). Second, the approach of concentra CA^21^ has been developed for toxicants with a similar effect mechanism. Hence, all toxicants in a mixture act as if they were a dilution of one another and the effect-normalized concentrations are added assuming an identical effect-normalized stressor-response relationship. The application of CA to the combined action of toxicant and environmental stressors also predicts an increase in toxicant sensitivity. However, this average increase is considerably below the observed shift in toxicant sensitivity and therefore also CA greatly under-predicts the observed joint mortality (Fig. 1B, solid lines). In contrast the SAM enables an effect prediction that approaches experimental reality much better although considerable variability remains to be explained. Both approaches, the CA and the SAM, have one aspect in common: normalized stress levels are added. However, the fundamental difference being that CA assumes a common stress-response relationship for both stressors. Accordingly, in instances where several stressors are characterised by similar effect mechanisms, we suggest using the traditional approach outlined by Loewe^21^. In contrast the SAM assumes independent stressors with specific stress-response relationships. The idea is that both stressors jointly reduce the common stress capacity of individuals. For toxicants, characterised by a right-skewed concentration-response relationship low toxicant concentrations disproportionally increase general stress compared to high toxicant concentrations. We argue that this relationship remains independent of the strength of the additional stressor.

The prerequisite of the SAM approach is that combined stressors are independent with respect to their mechanism of action. In the present meta-analysis, we postulate the following two domains of independent action: the first domain includes toxicants acting either on the signal transduction of individuals or exerting a general toxicity, for instance, through the formation of free radicals. The second domain includes environmental stressors that affect the general metabolic balance of an individual. In the present meta-analysis, the domain of environmental stressors included (i) food deficiency and competition for food; (ii) predator cues that have been linked to reduce the energy budget of individuals^22^ and (iii) two studies involving water level fluctuations and low oxygen concentrations, of which the effects on metabolic stress have not, to our knowledge, yet been examined. Accordingly, we assumed that the combined stressors of the studies generally act on different receptors within the organisms and are therefore considered independent in a first approximation. Within the current dataset this included toxicants on the one side and environmental stressors on the other side. However, also some toxicant combinations that act on different receptors within an organism may be considered independent and their combined action may be successfully predicted with the SAM. Therefore further work on categorizing stressors according to their respective domains of action is needed.

We expect the SAM approach to be a widely applicable starting point for assessing the direct effects of all types of multiple stressors. Based on the examples of the available experimental studies in the literature, it is clear that additional environmental stress strongly increases the toxicant sensitivity of individuals. The identification of such synergistic effects is highly relevant for an integrated environmental risk assessment, as stressors that alone may not exert measurable survival effects may magnify the effects of other stressors.

## Methods

### Meta-analysis on combined stressors

We compiled the available literature on the combined impacts of environmental stressors and toxicants on aquatic invertebrates. The relevant studies were identified by performing a search on ISI’s “Web of Science” using the following queries: (“multiple stress*” OR “combined stress*” OR “interactive effect*”) AND (“toxicant*” OR “insecticide*” OR “fungicide*” OR “herbicide*” OR “metal*”). This search, along with the literature already known to the authors, resulted in the identification of 23 pairs of concentration-response relationships involving 10 different taxa. As toxicant stressors, the selected studies included 4 organic insecticides, and 1 metal. As environmental stressors, we considered all natural factors that challenge an individual to expend additional resources such as energy. Hence, the selected studies comprised environmental stress caused by intraspecific and interspecific competition, food limitations, UV exposure, predator cues, water fluctuations and oxygen deficiency (for details, see Table S1). We excluded studies if the environmental stressor did not increase individual requirements, such as non-selective predation that randomly excludes individuals^23^. The endpoint of interest was the survival/mortality of organisms observed at the individual or population level; as such, sublethal endpoints were not considered. Studies were selected if (i) the concentration-response relationship of the toxicant was available for both experimental designs - with and without environmental stress and (ii) each of the two concentration-response relationships involved at least a control, a concentration with a partial response and a concentration providing a response close to 100% mortality. We only included studies that provided information on direct and acute toxicant effects as indirect effects can lead to less-than-additive, additive, or more-than-additive responses. Indirect effect not considered were (i) substantial recovery that reduced the combined stressor impact; (ii) population regulation through intraspecific competition that masked the combined effect of stressors^24^ or (iii) culmination of toxicant effects induced by sequential toxicant exposure^15^.

Survival data were extracted from the literature with the software “DigitizeIt” from Bormisoft, Braunschweig, Germany. Data were fitted applying the classic log-logistic model for concentration-response relationships to quantify the impact of toxicant with and without additional environmental stress^25^. We selected the five-parameter log-logistic function LL.5, which allows for an asymmetric fit of the data.


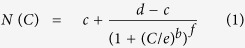


where the population size N is the proportion of surviving individuals depending on the toxicant concentration C; b > 0 represents the shape parameter; c and d are the lower and upper limits of N, respectively; e > 0 represents the scale parameter; and f is the parameter for asymmetry^25^. The lower limit c was fixed to 0, and the upper limit d was fixed to the population response value of the control. In addition, two data pre-processing steps were performed before applying the concentration-response model. First, several population-level studies indicate a slight hormesis effect in terms of a trend towards increasing survival at low concentration levels. For the purpose of simplification, we averaged effects of an increasing survival at higher concentrations by applying the Williams transformation^26^. Nevertheless, we believe that future work on combined stressor effects will benefit from considering hormesis, especially when a generally applicable mechanism for its prediction has been identified. Obviously compensation processes result in a reduced amplification of effects in one range of stressor combinations while increased amplification of effects occur in another range of the same stressor combinations. Second, a poor model fit was found when concentration-response models included only few data points with partial survival; a partial survival for a maximum of three concentrations was present in approximately 80% of the studies. Thus, to avoid manual fitting of each model or arbitrary setting of parameters. We used the statistical approach provided by the R-package (stats, function *approx*) to generate 10 smoothing data points by linear interpolation on a logarithmic scale. This approach resulted in an ecotoxicological reasonable model fit. The fitted curves are shown in Figure S1. From this we calculated the two lethal concentrations LC10 and LC50. The increase in toxicant sensitivity due to additional environmental stress is given as the shift of the respective LC, referred to here as LC10-shift (LC10/LC10*) and LC50-shift (LC50/LC50*). The level of environmental stress is estimated as the mortality caused by environmental stress alone. Table S1 summarizes information about the studies included, the level of environmental stress as well as the LC10-and LC50-shift.

### Mathematical description of the “Stress Addition Model” (SAM)

The 3 principal assumptions for the combined impact of independent stressors, in this case, toxicants and additional environmental stressors can be described as follows:

1. The first assumption of SAM is that each individual has a certain capacity to tolerate all types of stress, its general stress capacity. We assume this individual stress capacity to be symmetrically distributed over the finite interval [0, 1]. We argue that the stress-dependent population sensitivity follows the same distribution. Individuals with a stress capacity below a given stress level S will die, whereas individuals with a stress capacity above a given stress level will survive. Hence, the stress-dependent population sensitivity is parameterized by the following beta distribution

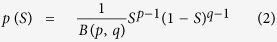


where p(S) represents the probability density of individuals to tolerate a general stress S, p and q as the non-negative shape parameters of the distribution and B(p, q) as the beta function which is a normalization constant to ensure that the total probability integrates to 1. We postulated symmetry of individual stress capacity (p = q). The parameters were set to p = q = 3.2 which resulted in the best fit between observed and predicted LC10 and LC50 shifts of the 23 experimental study-pairs (see Figure S2). Best model fit was determined by linear least-squares fitting which was applied on studies with an environmental stress mortality of greater than 0%. Goodness of fit was quantified as R^2^ that gives the proportion of variance explained by the model (shift of LC10: R^2^ = 0.49; shift of LC50: R^2^ = 0.38; see Figure S2).

The integral of the density function gives the population size N under non-stress conditions


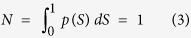


The stress-dependent survival is calculated as


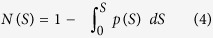


where N(S) = 1 (100% survival) for the general stress S = 0 and N(S) = 0 (0% survival) for the general stress S ≥ 1.

(2) The second assumption of the SAM is that every specific unit of a given stressor can be transferred into a general stress level. This conversion uses the stress-related mortality as a linking factor. For instance, if a temperature stress or toxicant stress causes a mortality of 10%, the general stress level is given by the 10% quantile of the beta distribution in Equation 2.

(3) The third assumption of SAM is that the general stress levels of independent stressors are additive, with the sum determining the total general stress exerted on a population. The total general stress S is given as the sum of general stress levels S_i_ of all independently acting stressors.


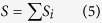


All studies of the meta-analysis combine a fixed environmental stress level S_ENV_ with an increasing toxicant stress level S_TOX_. Accordingly, the general stress S, calculated for an environmental stressor and a toxicant stressor, is thus expressed as follows:


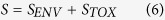


The resulting survival of the population exposed to the general stress S can be determined applying equation 4. Due to the low number of suitable studies (n = 23) we used the information of all experiments to parameterize the SAM. A validation of the approach requires additional experiments. The practical use of the SAM is facilitated by an Excel spreadsheet available as download within the SI (SAM_Calculator.xlsx).

The SAM combines empirical knowledge with mechanistic concepts in order to predict the combined effects of stressors. The empirical “share” comprises the parameterization of the beta distribution and also the parameterization of the average concentration-response relationship over all studies of the meta-analysis (Fig. 3). The mechanistic “share” of the SAM is based on the concept of subtracting general stress levels, the common currency of various stressors, from the individuals stress capacity as illustrated in Fig. 2.

### Model application for the meta-analysis

To predict the general impact of toxicant stress in the presence of environmental stress, an average concentration-response curve for toxicant stress was needed. For this we averaged all experimental concentration-response curves without environmental stress included in the meta-analysis. We normalized the test concentrations by restricting them to a range between the lethal concentrations LC1 and LC99 and scaled this concentration range to normalized values of 0 to 1 (Fig. 3A). This approach was chosen in order to approximate the shape of the concentration-response relationship and thus the underlying sensitivity distribution of populations towards toxicants. In a first step of averaging, we calculated the median and standard error at 10 equidistant concentration levels of the normalized curves. In a second step, median and standard errors were fitted using the five parameter log-logistic model (equation 1, Fig. 3A, red and light red lines). The resulting average concentration-response curve was then combined with increasing levels of environmental stress. The magnitude of the environmental stress was expressed by a stress-related mortality ranging from 1 to 90%. The increase of toxicant sensitivity due to environmental stress was quantified as the shift of LC10 (LC10/LC10*) and LC50 (LC50/LC50*). The calculation of the combined stress impact followed the instruction given in section ‘Mathematical description of the “Stress Addition Model” (SAM) and is available as an Excel spreadsheet within the SI (SAM_Calculator.xlsx).

Figures 3B,C illustrate the mortality-related link between the concentration levels of the averaged concentration-response curve and the general stress levels of the SAM. The relationship indicates that low toxicant concentrations disproportionally increase the general stress level compared to high toxicant concentrations.

### Traditional concepts of predicting stress combinations

**Figure 4:**
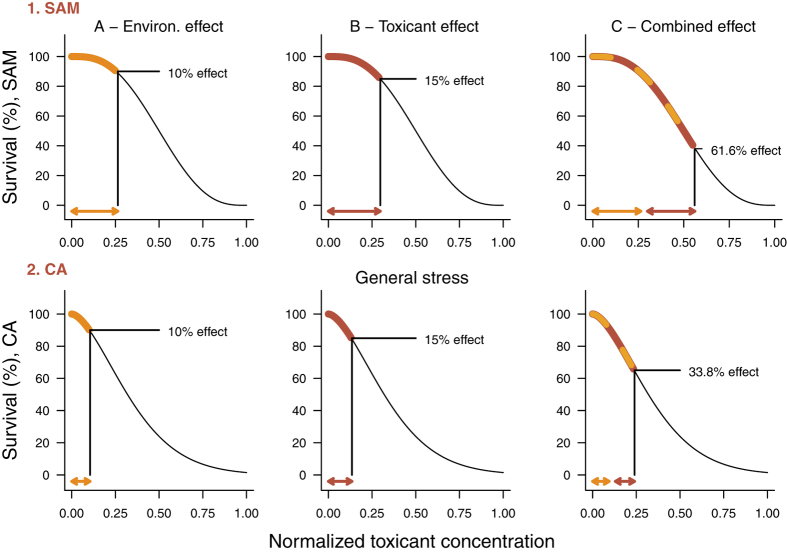
Comparison of the Stress Addition Model (SAM) and the Concentration Addition model (CA) based on the average normalized concentration-response curve. For SAM, the stress-response curve resulting from the beta distribution of the stress capacity is displayed. Mortality effects from the environmental stress (**A** −10%) and the toxicant stress alone (**B** −15%) have been converted into general stress levels that are presented as orange and red arrows. (**C**) The total stress that is exerted on a population is calculated as the sum of the stress levels of the independent stressors (orange and red arrows) and given as the combined effect (orange-red dashed line section). For CA, the average normalized concentration response curve of the toxicant is displayed. The mortality effect of the environmental stress alone was converted into the corresponding toxicant concentration (**A**, 10% effect; orange arrow). The toxicant effect (**B**, 15%) alone corresponds to the toxicant concentration represented by the red arrow. (**C**) The total effect on a population (orange and red dashed line section) is based on the sum of the normalized toxicant concentrations (orange and red arrow).

We compared the result obtained by the SAM with the predictions of the two traditional model concepts for the combined effect assessment of toxicants in pharmacology, namely, the effect addition model EA^20^ and the concentration addition model CA^21^. A comparison of the approach of the SAM and the adapted CA is shown in Fig. 4. In this example, the environmental stress causes a mortality of 10% and is combined with a toxicant stress causing a mortality of 15%. For SAM, these mortalities translate into general stress levels of 0.26 and 0.30 respectively. Accordingly the combined effects of both stressors add to a comparable high mortality of 61.6% (Fig. 4C). In contrast, for the CA model, the mortality exerted by the two stressors (10%, 15%) resulted in a much lower combined mortality of 33.8%.

To calculate this example, the traditional CA approach of pharmacology had to be adapted. The traditional CA approach assumes identical concentration-response relationships for all toxicants of a given mixture. Accordingly we adapted the CA approach by assuming identical stress-response relationships for the environmental and toxicant stressor of our studies. In the example above, the toxicant stress was quantified by the average normalized concentration-response relationship over all individual studies (see Fig. 3A). As in the SAM, we used the mortality rate as a link: The environmental stress that induced a mortality of 10% corresponds to the normalized toxicant concentration of 0.1 of the common concentration-response relationship. Finally, the two concentration values were added leading to the joint effect of 33.8% mortality (Fig. 4).

In the present study, the adapted CA approach was applied twice. First, we applied CA on the average normalized concentration-response relationship over all individual studies (see Fig. 3A) combined with an increasing level of environmental stress which results in the overall prediction of Fig. 1B. Second, we applied CA on the data of all individual studies of the meta-analysis which leads to the model fit depicted in Figure S3. Both applications show that the adapted CA approach predicts an increase of population sensitivity in the presence of environmental stress as well, however to a much lesser extent as predicted with the SAM approach.

For the approach of EA the effect is added according to the equation by Bliss^20^,


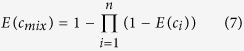


where E(c_mix_) is the total effect of the single effects of all stressors E(c_i_). For our example we calculate E(c_mix_) = 1 − (1 − 0.10)*(1 − 0.15) = 0.235. Hence, with the EA model in our example we only predict a mortality of 23.5% as the combined effect of independently acting stressors. For the general prediction, at all levels of the environmental stressor, the population sensitivity to toxicant remains unchanged. This independence of the toxicant sensitivity is in accordance with the definition of EA that assumes no interaction of sensitivities between both stressors. Hence, EA predicts no shift of population sensitivity and no synergism of the given stressor combination (Fig. 1B).

All statistical analyses were performed with R, version 3.1.2 (R Foundation for Statistical Computing, 2014, Vienna, Austria).

## Additional Information

**How to cite this article**: Liess, M. *et al*. Predicting the synergy of multiple stress effects. *Sci. Rep.* **6**, 32965; doi: 10.1038/srep32965 (2016).

## Supplementary Material

Supplementary Information

Supplementary Information 1

## References

[b1] Agency E. E. The European environment - state and outlook 2015: synthesis report. (Publications Office of the European Union, 2015).

[b2] Stehle S. & Schulz R. Agricultural insecticides threaten surface waters at the global scale. Proceedings of the National Academy of Sciences of the United States of America 112, 5750–5755, 10.1073/pnas.1500232112 (2015). 25870271 PMC4426442

[b3] Liess M. & Von Der Ohe P. C. Analyzing effects of pesticides on invertebrate communities in streams. Environ Toxicol Chem 24, 954–965 (2005). 15839571 10.1897/03-652.1

[b4] Schäfer R. B. . Thresholds for the Effects of Pesticides on Invertebrate Communities and Leaf Breakdown in Stream Ecosystems. Environ. Sci. Technol. 46, 5134–5142, 10.1021/es2039882 (2012). 22455566

[b5] Beketov M. A., Kefford B. J., Schafer R. B. & Liess M. Pesticides reduce regional biodiversity of stream invertebrates. Proceedings of the National Academy of Sciences of the United States of America 110, 11039–11043, 10.1073/Pnas.1305618110 (2013). 23776226 PMC3704006

[b6] Poteat M. D. & Buchwalter D. B. Four reasons why traditional metal toxicity testing with aquatic insects is irrelevant. Environ Sci Technol 48, 887–888, 10.1021/es405529n (2014). 24372053

[b7] Garnier-Laplace J. . Are radiosensitivity data derived from natural field conditions consistent with data from controlled exposures? A case study of Chernobyl wildlife chronically exposed to low dose rates. J Environ Radioact 121, 12–21, 10.1016/j.jenvrad.2012.01.013 (2013). 22336569

[b8] Holmstrup M. . Interactions between effects of environmental chemicals and natural stressors: A review. Science of the Total Environment 408, 3746–3762, 10.1016/J.Scitotenv.2009.10.067 (2010). 19922980

[b9] Pettis J. S., vanEngelsdorp D., Johnson J. & Dively G. Pesticide exposure in honey bees results in increased levels of the gut pathogen Nosema. Naturwissenschaften 99, 153–158, 10.1007/s00114-011-0881-1 (2012). 22246149 PMC3264871

[b10] Henry M. . Pesticide risk assessment in free-ranging bees is weather and landscape dependent. Nat Commun 5, 4359, 10.1038/ncomms5359 (2014). 25008773

[b11] Liess M., Champeau O., Riddle M., Schulz R. & Duquesne S. Combined effects of ultraviolett-B radiation and food shortage on the sensitivity of the Antarctic amphipod Paramoera walkeri to copper. Environ. Toxicol. Chem. 20, 2088–2092 (2001). 11521839 10.1002/etc.5620200931

[b12] Rohr J. R. . Agrochemicals increase trematode infections in a declining amphibian species. Nature 455, 1235–1239, 10.1038/nature07281 (2008). 18972018

[b13] Gordon C. J., Johnstone A. F. M. & Aydin C. Thermal Stress and Toxicity. Compr Physiol 4, 995–1016, 10.1002/cphy.c130046 (2014). 24944028

[b14] Henry M. . A Common Pesticide Decreases Foraging Success and Survival in Honey Bees. Science 336, 348–350, 10.1126/science.1215039 (2012). 22461498

[b15] Liess M. . Culmination of low-dose pesticide effects. Environ Sci Technol 47, 8862–8868, 10.1021/es401346d (2013). 23859631 PMC3781603

[b16] Segner H., Schmitt-Jansen M. & Sabater S. Assessing the Impact of Multiple Stressors on Aquatic Biota: The Receptor’s Side Matters. Environ Sci Technol 48, 7690–7696, 10.1021/es405082t (2014). 24905720

[b17] Goulson D., Nicholls E., Botias C. & Rotheray E. L. Bee declines driven by combined stress from parasites, pesticides, and lack of flowers. Science 347, 1255957, 10.1126/science.1255957 (2015). 25721506

[b18] Crain C. M., Kroeker K. & Halpern B. S. Interactive and cumulative effects of multiple human stressors in marine systems. Ecology Letters 11, 1304–1315, 10.1111/J.1461-0248.2008.01253.X (2008). 19046359

[b19] Van der Geest H. G., Soppe W. J., Greve G. D., Kroon A. & Kraak M. H. S. Combined effects of lowered oxygen and toxicants (copper and diazinon) on the mayfly Ephoron virgo. Environ. Toxicol. Chem. 21, 431–436 (2002). 11837232 10.1897/1551-5028(2002)021<0431:ceoloa>2.0.co;2

[b20] Bliss C. I. The toxicity of poisons applied jointly. Annals of Applied Biology 26, 585–615 (1939).

[b21] Loewe S. & Muischnek H. In Naunyn-Schmiedebergs Arch Exp Pathol Pharmakol Vol. 114, 313–326 (1926).

[b22] Janssens L. & Stoks R. Predation risk causes oxidative damage in prey. Biol Letters 9, 20130350, 10.1098/Rsbl.2013.0350 (2013). PMC3730648 23760170

[b23] Beketov M. A. & Liess M. The influence of predation on the chronic response of Artemia sp populations to a toxicant. Journal of Applied Ecology 43, 1069–1074, 10.1111/J.1365-2664.2006.01226.X (2006). 18784796 PMC2368765

[b24] Postma J. F., Buckert-de Jong M. C., Staats N. & Davids C. Chronic toxicity of cadmium to Chironomus riparius (Diptera: Chironomidae) at different food levels. Arch Environ Contam Toxicol 26, 143–148 (1994). 8311506 10.1007/BF00224797

[b25] Ritz C. & Streibig J. C. Bioassay analysis using R. Journal of Statistical Software 12, 1–22 (2005).

[b26] Williams D. A. Test for Differences between Treatment Means When Several Dose Levels Are Compared with a Zero Dose Control. Biometrics 27, 103-&, 10.2307/2528930 (1971). 5547548

