# Supplementary material for: Predicting the synergy of multiple stress effects
Source: Sci Rep. 2016 Sep 9;6:32965. doi: 10.1038/srep32965 (PMC5017025; doi:10.1038/srep32965)
Supplement: Supplementary Information [file srep32965-s1.pdf]

Supplementary Information

Predicting the synergy of multiple stress effects

Matthias Liess,[1,2]* Kaarina Foit,[1] Saskia Knillmann,[1] Ralf B. Schäfer,[3] Hans-Dieter Liess[4]

Affiliation:

[1] UFZ, Helmholtz Centre f. Environmental Research, Dept. System-Ecotoxicology, Permoserstr. 15, 04318 Leipzig, Germany

[2] RWTH Aachen University, Institute for Environmental Research (Biology V), Worringerweg 1, 52074 Aachen, Germany

[3] University Koblenz-Landau, Institute for Environmental Sciences, Fortstrasse 7, 76829 Landau in der Pfalz, Germany

[4] University of the Bundeswehr München, Faculty EIT, Werner-Heisenberg-Weg 39, 85577 Neubiberg, Germany

*Correspondence to: matthias.liess@ufz.de

Table S1 | List of studies included for meta-analysis. Stress determined as the difference between control survival without and with environmental stress (Environment. stressor-mortality (%), based on fitted curves, see Figure S1) and sensitivity data (LC10 and LC50 using log-logistic concentration-response curves). LC10-Shift and LC50-Shift calculated as the concentration of LCx without environmental stress divided by the concentration of LCx* with environmental stress. MoA (Mode of Action): VGSC - voltage gated sodium channel modulator; FFR - formation free radicals, reducing reactive oxygen species; AChE - acetylcholinesterase inhibitor. The meta-analysis comprised 23 pairs of concentration-response relationships. These pairs included 6 different environmental stressors, 5 toxicants and 10 species.

Study ID	Reference	Species	Toxicant - substance	Toxicant - MoA	Toxicant - type of exposure	Environmental stressor - type	Environment. stressor - mortality (%)	Time after toxic. exposure (d)	LC10-Shift	LC50-Shift
1	27	*Daphnia magna*	Fenvalerate	VGSC mod.	pulse	Food limitation	0.0	8	2.1	1.8
2	28	*Daphnia magna*	Fenvalerate	VGSC mod.	pulse	Maternal food	0.0	7	3.1	2.6
3	29	*Bufo americanus*	Malathion	AChE inhi.	continuous	Predator cues	0.0	10	1.3	1.1
4	29	*Rana pipiens*	Malathion	AChE inhi.	continuous	Predator cues	0.0	7	1.2	1.5
5	19	*Ephoron virgo*	Copper	FFR, RROS	continuous	Low oxygen	0.0	4	5.0	2.0
6	30	*Cloeon dipterum*	Esfenvalerate	VGSC mod.	pulse	Food limitation	0.0	8	4.7	1.4
7	29	*Rana sylvatica*	Malathion	AChE inhi.	continuous	Predator cues	0.0	5	1.1	1.0
8	31	*Rana pipiens*	Carbaryl	AChE inhi.	continuous	Predator cues	0.0	8	1.4	1.1
9	11	*Paramoera walkeri*	Copper	FFR, RROS	continuous	Food limitation	0.6	21	1.2	1.1
10	31	*Rana clamitans*	Carbaryl	AChE inhi.	continuous	Predator cues	1.0	9	7.6	3.7
11	31	*Rana catesbeiana*	Carbaryl	AChE inhi.	continuous	Predator cues	1.0	7	3.6	1.9
12	31	*Bufo americanus*	Carbaryl	AChE inhi.	continuous	Predator cues	1.5	9	4.0	1.4
13	29	*Rana clamitans*	Malathion	AChE inhi.	continuous	Predator cues	2.4	12	1.0	1.3
14	31	*Hyla versicolor*	Carbaryl	AChE inhi.	continuous	Predator cues	2.5	7	1.0	1.2
15	29	*Rana catesbeiana*	Malathion	AChE inhi.	continuous	Predator cues	6.1	7	0.4	0.9
16	30	*Cloeon dipterum*	Esfenvalerate	VGSC mod.	pulse	Food limitation	6.7	8	12.9	2.0
17	29	*Hyla versicolor*	Malathion	AChE inhi.	continuous	Predator cues	13.3	10	5.1	2.2
18	11	*Paramoera walkeri*	Copper	FFR, RROS	continuous	Food limitation	13.9	21	13.0	12.5
19	32	*Daphnia* spp.	Esfenvalerate	VGSC mod.	pulse	Intrasp. compet.	22.8	4	3.6	1.6
20	33	*Hyla versicolor*	Carbaryl	AChE inhi.	continuous	Predator cues	24.5	16	17.6	7.7
21	34	*Daphnia* spp.	Esfenvalerate	VGSC mod.	pulse	Water level alt.	37.7	8	26.4	17.3
22	32	*Daphnia* spp.	Esfenvalerate	VGSC mod.	pulse	Intrasp. compet.	42.1	4	51.8	5.9
23	11	*Paramoera walkeri*	Copper	FFR, RROS	continuous	UV radiation	47.9	21	140.8	42.1

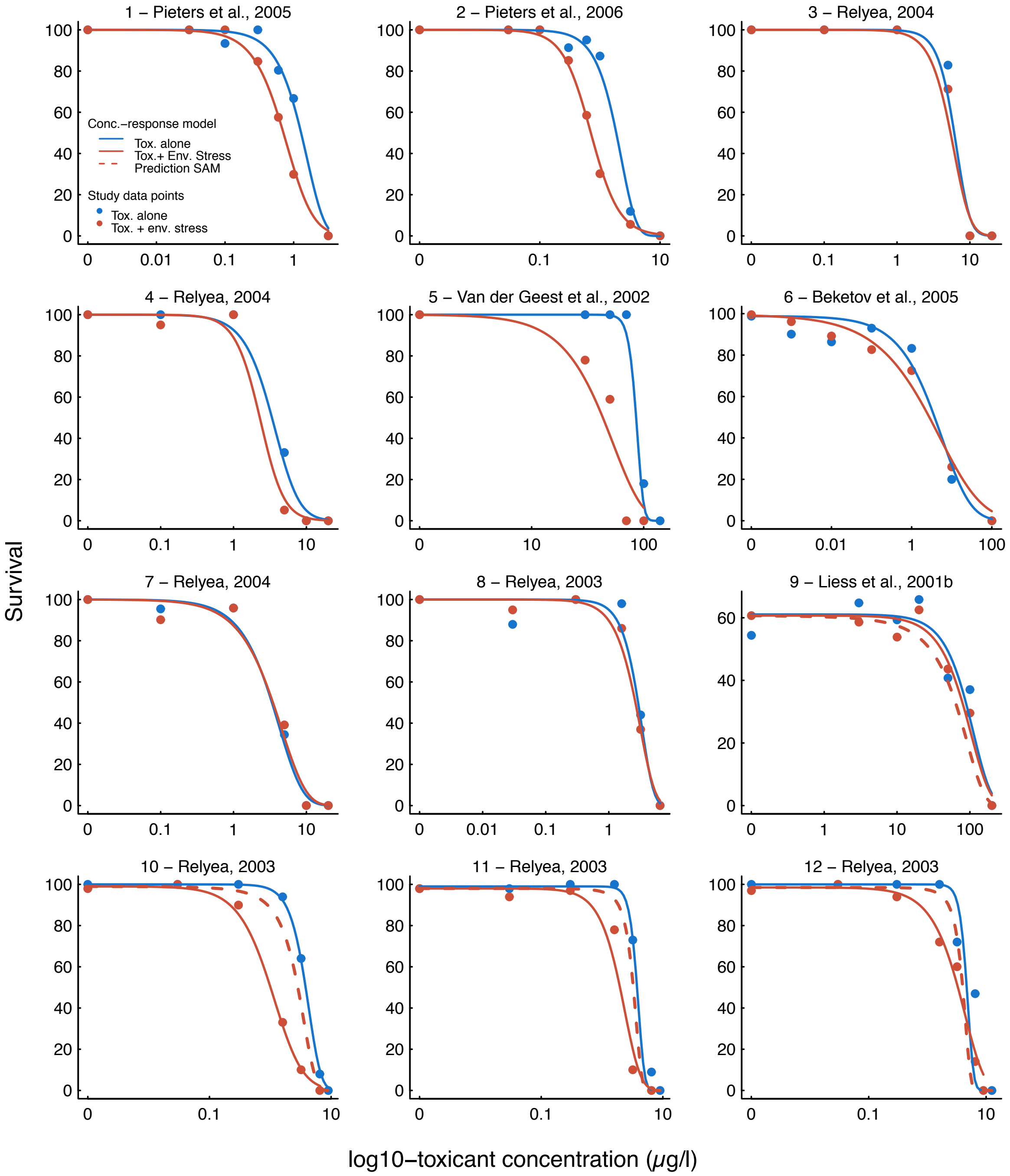

Figure S1 | Survival data from the meta-analysis. — Continued on next page —

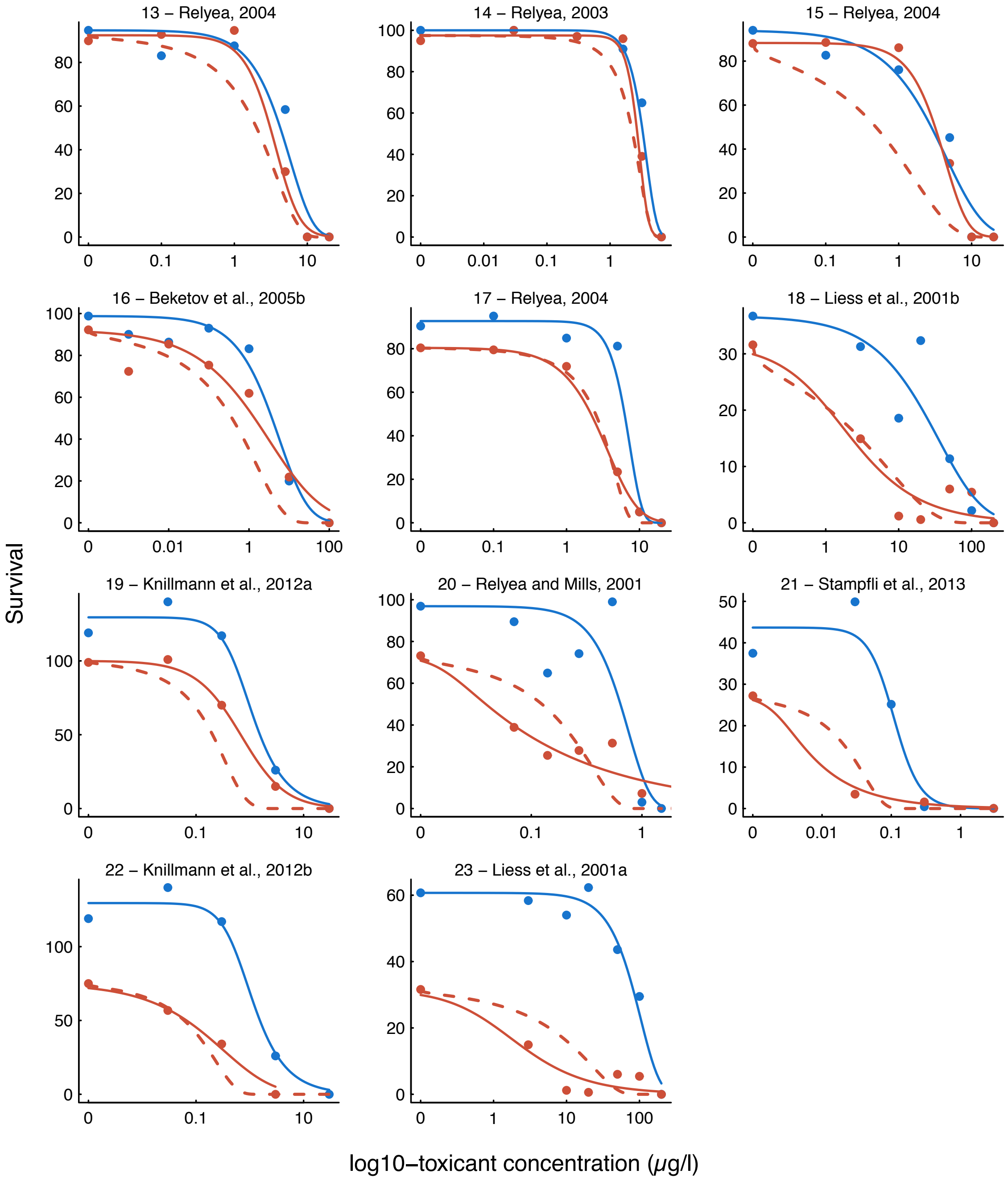

Figure S1 | Survival data from the meta-analysis. Concentration-response relationships observed from empirical studies. Pairs of concentration-response relationships are displayed - without environmental stress (blue points, line) and with environmental stress (red points, line). Survival is either given as % survival of the control or as number of individuals, as provided from the original publication. The red dashed line represents the modelled concentration-response relationship under environmental stress using the SAM approach. The SAM modelling was only performed when the environmental stress resulted in mortality greater than 1%.

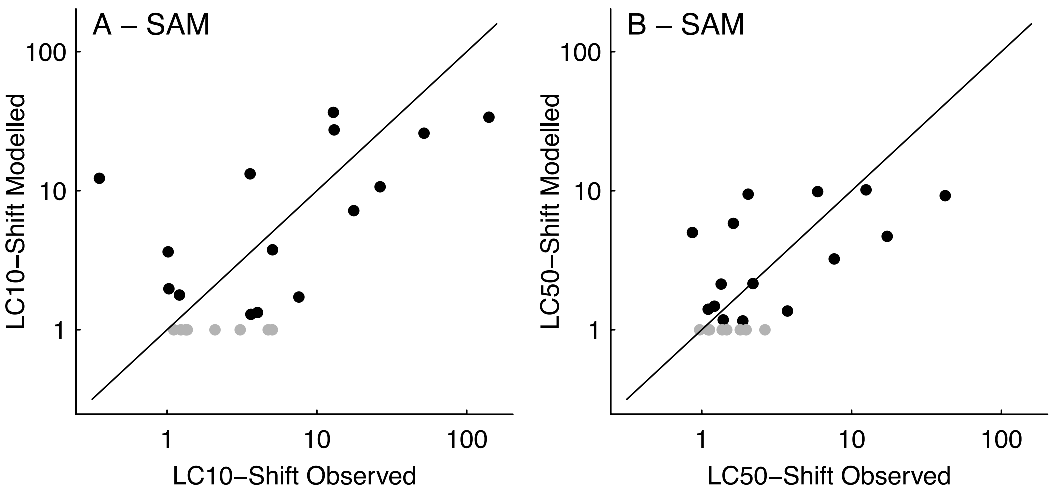

Figure S2 | SAM-modelled shifts of LC10 and LC50 in comparison to observations based on the study specific concentration-response curves from each of the 23 studies in the meta-analysis. The toxicant sensitivity is expressed as shift of the LCx (A: LC10/LC10*; B: LC50/LC50*). The environmental stress of studies resulted in a mortality greater than 0 % (black points) or equal to 0 % (grey points). The diagonal lines represent the ideal correlation (45° line). Goodness of fit was quantified as R^2 that gives the proportion of variance explained by the model (A: $R^2 = 0.49$; B: $R^2 = 0.38$). For quantification we considered only those studies with observed environmental stress mortality greater than 0% (black points) and positive shifts of LC10 and LC50.

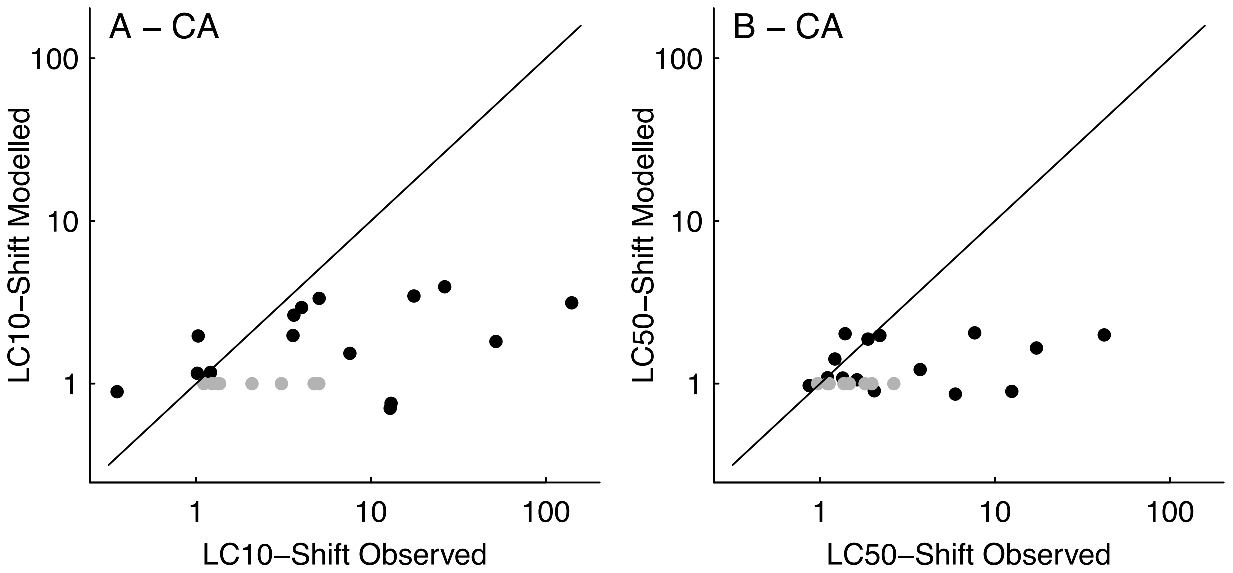

Figure S3 | CA-modelled shifts of LC10 and LC50 in comparison to observations based on the study specific concentration-response curves from each of the 23 studies in the meta-analysis. The toxicant sensitivity is expressed as shift of the LCx (A: LC10/LC10*; B: LC50/LC50*). The environmental stress of studies resulted in a mortality greater than 0 % (black points) or equal to 0 % (grey points). The diagonal lines represent the ideal correlation (45° line). Goodness of fit was quantified as R^2 that gives the proportion of variance explained by the model (A: $R^2 = -0.88$; B: $R^2 = -0.77$; with negative R^2 indicating that CA is better explained by the average of observations). For quantification we considered only those studies with observed environmental stress mortality greater than 0% (black points) and positive shifts of LC10 and LC50.

References:

27 Pieters, B. J. *et al.* Influence of food limitation on the effects of Fenvalerate pulse exposure on the life history and population growth rate of Daphnia magna. *Environmental Toxicology and Chemistry* **24**, 2254-2259 (2005).

28 Pieters, B. J. & Liess, M. Maternal nutritional state determines the sensitivity of Daphnia magna offspring to short-term Fenvalerate exposure. *Aquat Toxicol* **76**, 268-277, doi:10.1016/j.aquatox.2005.09.013 (2006).

29 Relyea, R. A. Synergistic impacts of malathion and predatory stress on six species of North American tadpoles. *Environ Toxicol Chem* **23**, 1080-1084, doi:10.1897/03-259 (2004).

30 Beketov, M. A. & Liess, M. Acute contamination with esfenvalerate and food limitation: chronic effects on the mayfly, Cloeon dipterum. *Environ Toxicol Chem* **24**, 1281-1286 (2005).

31 Relyea, R. A. Predator cues and pesticides: A double dose of danger for amphibians. *Ecological Applications* **13**, 1515-1521 (2003).

32 Knillmann, S., Stampfli, N. C., Beketov, M. A. & Liess, M. Intraspecific competition increases toxicant effects in outdoor pond microcosms. *Ecotoxicology* **21**, 1857-1866, doi:10.1007/s10646-012-0919-y (2012).

33 Relyea, R. A. & Mills, N. Predator-induced stress makes the pesticide carbaryl more deadly to gray treefrog tadpoles (Hyla versicolor). *Proceedings of the National Academy of Sciences of the United States of America* **98**, 2491-2496, doi:Doi 10.1073/Pnas.031076198 (2001).

34 Stampfli, N. C. *et al.* Two stressors and a community: effects of hydrological disturbance and a toxicant on freshwater zooplankton. *Aquat Toxicol* **127**, 9-20, doi:10.1016/j.aquatox.2012.09.003 (2013).
